# Tissue-Specific Metabolite Profiling on the Different Parts of Bolting and Unbolting *Peucedanum praeruptorum* Dunn (Qianhu) by Laser Microdissection Combined with UPLC-Q/TOF–MS and HPLC–DAD

**DOI:** 10.3390/molecules24071439

**Published:** 2019-04-11

**Authors:** Ling Li Chen, Shan Shan Chu, Ling Zhang, Jin Xie, Min Dai, Xin Wu, Hua Sheng Peng

**Affiliations:** 1 College of Pharmacy, Anhui University of Chinese Medicine, Hefei 230012, China; chenll0421@126.com (L.L.C.); cshan0916@126.com (S.S.C.); daiminliao@163.com (M.D.); wuxinang@163.com (X.W.); 2 College of Pharmacy, Anhui Medical University, Hefei 230032, China; xiejin159@126.com; 3 Synergetic Innovation Center of Anhui Authentic Chinese Medicine Quality Improvement, Hefei 230038, China

**Keywords:** bolting, *Peucedanum praeruptorum* Dunn, microstructure, LMD, UPLC-Q/TOF–MS, HPLC–DAD

## Abstract

Background: Qianhu is a traditional Chinese medicine. It is thought that Qianhu roots will harden after bolting and not be suitable for medicinal purposes. Bolting Qianhu and unbolting Qianhu are referred to as “Xiong Qianhu” and “Ci Qianhu,” respectively. In this study, the properties, microscopic and chemical characteristics of Ci Qianhu and Xiong Qianhu roots were compared using fluorescence microscopy, laser microdissection coupled with ultra-high-performance liquid chromatography-quadrupole/time-of-flight mass spectrometry, and high-performance liquid chromatography with diode-array detection. Results: Microscopy results showed that the area of secondary xylem in the root increased after bolting, with the cork and secretory canals showing strong fluorescence intensity. A total of 34 peaks, mostly pyranocoumarins, were identified in the tissues of Ci Qianhu and Xiong Qianhu. The secretory canals contained the highest variability of coumarins, whereas the secondary xylem contained the least coumarins. Moreover, seven coumarins, especially the pyran- coumarin, decreased after bolting. Generally, both before and after bolting, coumarin level was the highest in the bark, followed by the middle part, and the lowest in the inner part. Conclusion: Thus, it was indicated that the area of secondary xylem increased after bolting, however the coumarin variant and content decreased in the secondary xylem of Qianhu. The result shows that the quality of Qianhu decreases after bolting, which supports the viewpoint that Xiong Qianhu is not suitable for medicinal use.

## 1. Introduction

Peucedani radix (Qianhu in Chinese), a well-known traditional Chinese medicine, was first recorded in *MING YI BIE LU*, an ancient herbal book over 1500 years old. In the time immemorial, Qianhu was used to cure cough and expectoration of thick yellow mucus, lung diseases, and phlegm accumulation in the respiratory tract [1]. According to the Chinese Pharmacopoeia (2015 Edition), peucedani radix is the dried root of unbolting or no-flowering *Peucedanum praeruptorum* Dunn. The clinical application of Qianhu has been increasingly extensive due to its status as one of the main ingredients in many Chinese patent drugs. Qianhu has also been traditionally used for medicinal purposes in other Asian countries, such as Korea and Japan, where it is known as “Jeonho” [2] and “Zenko” [3], respectively.

**Figure 1:**
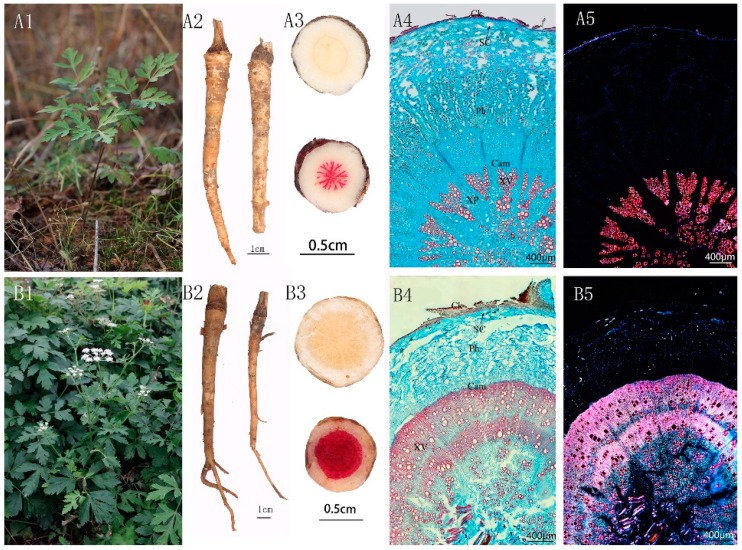
Macroscopic and microscopic morphology of Qianhu. (A: Sample1; B: Sample2) (**A1**) the Raw plants of Ci Qianhu;(**A2**) the medicinal materials of Ci Qianhu; (**A3**) the cross-section stained with phloroglucinol and concentrated hydrochloric acid of Ci Qianhu; (**A4**) observed under a normal light microscope of Ci Qianhu; (**A5**) observed under polarized light of Ci Qianhu; (**B1**) the Raw plants of Xiong Qianhu; (**B2**) the medicinal materials of Xiong Qianhu; (**B3**) the cross-section stained with phloroglucinol and concentrated hydrochloric acid of Xiong Qianhu; (**B4**) observed under a normal light microscope of Xiong Qianhu; (**B5**) observed under polarized light of Xiong Qianhu. CK, cork; Ph, phloem; SC, secretory canals; Cam, cambium; XV, xylem vessels; XR, xylem ray.

Qianhu is a flowering plant which blooms only once in its lifetime. Qianhu can grow in the wild without blooming for years, whereas cultivated Qianhu usually bears fruit after only 1–2 years of growth. The root started to lignify after bolting, which hardened it and decreased its weight. Thus, it was traditionally believed that bolting Qianhu could not be used as medicine. In the horticulture field, bolting Qianhu and unbolting Qianhu are referred to as “Xiong Qianhu” (Figure 1B) and “Ci Qianhu” (Figure 1A), respectively. The view that “Xiong Qianhu” are not suitable for medicinal purposes has been emphasized by the herbalists from the Qing Dynasty [4] and inherited by modern medical practitioners.

Modern pharmacological studies have reported the anti-inflammatory [5,6], anti-osteoporosis [7], hypotensive, coronary dilatory, anti-myocardial dysfunction [8], anti-cancer [9], and acute lung injury-relieving activities of Qianhu [10]. Coumarins have been identified as the main active ingredients of Qianhu, particularly angular-type pyranocoumarins such as praeruptorin A, praeruptorin B, and praeruptorin E [11]. It is important to examine the changes in the chemical composition of Qianhu before and after bolting.

Qianhu is widely distributed in many areas of China. Cultivation of Qianhu has been increasing in recent years because of the depletion of natural resources and the increase in demand. Ningguo County, Anhui Province is the original cultivation site of Qianhu, which contributes one-third of the national annual export and sales of Qianhu. To evaluate the quality of Ci Qianhu and Xiong Qianhu, the relative contents of several coumarins and volatile oil have been comparatively studied [12,13,14]. These studies suggested that the change of the proportion of the main components in Qianhu before and after bolting may be the reason made Qianhu incapable for medicinal use. However, these studies on the accumulation of coumarins were limited to the relative quantification of several components based on the crushing and extraction of intact roots, there is scarcely any information on the comprehensive comparison of metabolites and structure in Ci Qianhu and Xiong Qianhu. Laser microdissection (LMD) is an accurate and simple technology for separating the specific single type of tissues or individual cells under the microscope, which has been successfully applied to reveal the spatial distribution of metabolites in some medicinal herbs [15,16,17]. In this study, LMD combined with ultra-performance liquid chromatography-quadrupole-time of flight-mass spectrometry (UPLC-Q-TOF–MS) was used to elucidate the distribution pattern of chemical compounds in the tissues to evaluate the relationship between structure and chemical composition of Xiong Qianhu and Ci Qianhu. In addition, HPLC–DAD was used to analyze quantitatively the chemical constituents of different parts of Ci Qianhu and Xiong Qianhu. Thus, these qualitative and quantitative data were used to empirically clarify the suitability of Xiong Qianhu as medicine.

## 2. Results

### 2.1. The Characteristics and Microscopic Frameworks Between Ci Qianhu and Xiong Qianhu

#### 2.1.1. Ci Qianhu

The root of Ci Qianhu was fleshy, irregularly cylindrical or spindle-shaped, and slightly curved at the lower part with some prominent scars of rootlets. The epidermis was brown, with fine ring lines densely covering the root cap and transverse lenticel-like protrudins at the root (see Figure 1A2). The roots were relatively soft with strong fragrance. White phloem, yellow xylem, and yellowish-brown cambium ring were observed in the cross-section of the roots. With phloroglucinol and concentrated hydrochloric acid staining, the xylem of the cross-section was red and radiate, with wider rays (see Figure 1A3). The anatomical structure of Qianhu root was comprised of cork, secondary phloem, cambium, and secondary xylem from the periphery to the center. The secondary phloem was broad and scattered, with abundant brown secretory canals, and fracture and starch grains distributed in the phloem parenchymal cells. The secondary xylem was narrow, accounted for 20% of the root, and was composed of vessels, parenchymal cells, and rays. Numerous vessels were closely gathered, and the vessel bundles were arranged radially (see Figure 1A4,A5).

#### 2.1.2. Xiong Qianhu

Xiong Qianhu root was irregularly cylindrical or spindle-shaped, with prominent scars of rootlets. The epidermis was brown, with stem scars remaining at the root head (see Figure 1B2). The roots were relatively hard, with an insipid odor. Phloroglucinol and concentrated hydrochloric acid staining revealed red in xylem with more thickened parenchymal cells than those of Ci Qianhu (see Figure 1B3). Compared with Ci Qianhu, the secondary phloem was narrow, with larger secretory canals, more fracture, and fewer starch grains in the secondary phloem. The secondary xylem was broad, with a larger area than that of secondary phloem, which accounted for over 50% of the whole area. The vessels were arranged loosely and scattered in the secondary xylem (see Figure 1B4,B5).

Therefore, the differences in appearance between Ci Qianhu and Xiong Qianhu are the texture and the smell. The root of Ci Qianhu is fleshy, but the root of Xiong Qianhu is skinny with it lignified. Therefore, the texture of Ci Qianhu is soft, while Xiong Qianhu is as hard as wood. Qianhu is an Umbelliferae plant with a very strong aroma. And after bolting, the fragrance of Qianhu tends to fade. In microscopic form, the difference between the two is reflected in the thickening of xylem parenchyma cells and the proportion of xylem area. The xylem parenchyma of Xiong Qianhu is thicker and the area of the xylem is increased, which leads to phloem rupture.

### 2.2. Separation of Different Cells and Tissues by Fluorescence Microscopy 

**Figure 2:**
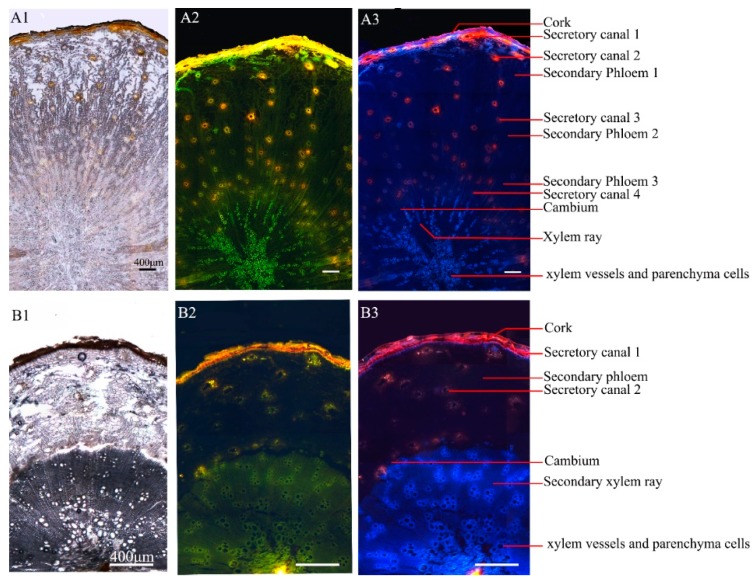
Microscopic characteristics of different parts of Qianhu. (A: Sample 8; B: Sample 4) (**A1**) observed under normal light mode of Ci Qianhu; (**A2**) observed under fluorescence mode of Ci oianhu (excitation wavelength: mixed 450–490 nm and 460–500 nm); (**A3**) observed under fluorescence mode of Ci Qianhu (equipped with triple band filter RGB); (**B1**) observed under normal light mode of Xiong Qianhu. (**B2**) observed under fluorescence mode of Xiong Qianhu (excitation wavelength: mixed 450–490 nm and 460–500 nm); (**B3**) observed under fluorescence mode of Xiong Qianhu (equipped with triple band filter RGB).

The frozen section of Ci Qianhu and Xiong Qianhu was observed in the bright field (see Figure 2A1,B1), some differences in the cork, the secondary phloem, the secondary xylem, and the secretory canals of Ci Qianhu and Xiong Qianhu could be seen from the color and size. The main active compounds of Qianhu are coumarins, which often exhibit different fluorescence at different excitation wavelengths [18]. Therefore, fluorescence microscopy is used for examining the distribution of coumarins in various tissues of Qianhu. The result showed a clear distinction under the two fluorescent mixtures with an excitation wavelength of 450–490 nm and 460–500 nm. In this way, the differences between different tissues can be observed more clearly instead of displaying a single color. The yellow fluorescence was emitted from the cork, while the light green color appeared in the secondary phloem, and the xylem vessels showed green fluorescence. The secretory canals of the secondary phloem showed fluorescence, and different secretory canals presented an obvious difference in fluorescence intensity. The bright orange-yellow color was shown in secretory canals of larger diameter near cork, orange or light green color was presented in secretory canals of smaller diameter at middle of secondary phloem while the green color was shown in secretory canals of more quantities but smallest diameter near the cambium (Figure 2A2). Then, the cork, secondary phloem (divided into three parts), cambium, secondary xylem, and secretory canals (divided into four types) of Ci Qianhu were separated by LMD based on their fluorescence characteristics.

Different tissues of Xiong Qianhu also showed different fluorescence under the same fluorescence mode. The main differences were that the deeper orange-yellow color in the cork of Xiong Qianhu was shown compared to Ci Qianhu; both the secondary phloem and secondary xylem of Xiong Qianhu presented a green color, and its secondary xylem vessels showed weaker fluorescence compared with Ci Qianhu. The secretory canals in Xiong Qianhu were less than that of Ci Qianhu, but bigger in diameters. The secretory canals near cork showed bright yellow color while that in secondary phloem presented green color (Figure 2B2). Moreover, the similar fluorescence characteristics were observed in Ci Qianhu and Xiong Qianhu by LMD, which equipped with the triple band filter RGB (Figure 2A3,A4). Similarly, the cork, secondary phloem, cambium, secondary xylem, and secretory canals (divided into two types) of Xiong Qianhu were separated based on their fluorescence characteristics.

### 2.3. Metabolite Profiling by UPLC-Q/TOF–MS 

**Figure 3:**
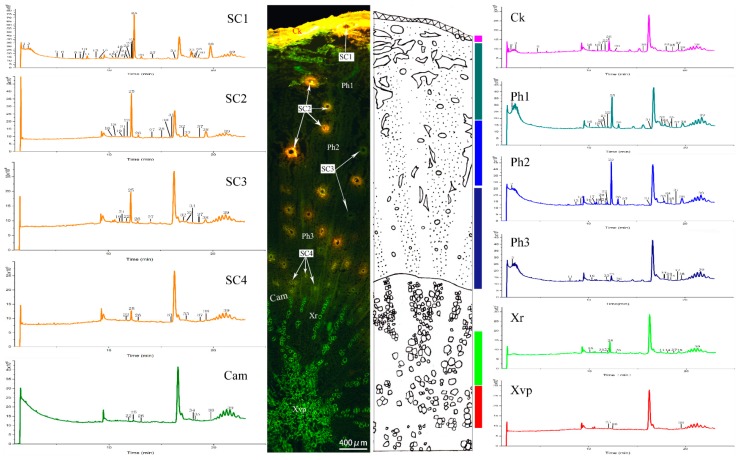
LC–MS base peak chromatograms of microdissected tissues from Ci Qianhu (Sample 8). The peak numbers referred to Table 1. SC1, secretory canals 1; SC2, secretory canals 2; SC3, secretory canals 3; SC4, secretory canals 4; Cam, cambium; Ck, cork; Ph1, secondary phloem1; Ph2, secondary phloem2; Ph3, secondary phloem3; Xr, xylem ray; Xvp, xylem vessels and parenchyma cells.

**Figure 4:**
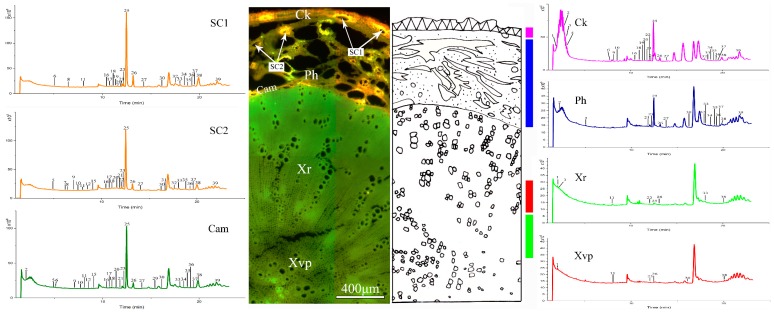
LC–MS base peak chromatograms of microdissected tissues from Xiong Qianhu (Sample 9). The peak numbers referred to Table 1. SC1, secretory canals 1; SC2, secretory canals 2; Cam, cambium; CK, cork; Ph, secondary phloem; XR, xylem ray; Xvp, xylem vessels and parenchyma cells.

The dissected tissues of Ci Qianhu and Xiong Qianhu were analyzed by UPLC-Q/TOF–MS method established above and sample 3 to sample 12 were used for this experiment. Among these samples, there were 5 Ci Qianhu and 5 Xiong Qianhu. The number of peaks measured in different tissues of each sample is different. However, the distribution pattern of the compounds is similar. The total ions current (TIC) chromatograms of microdissected tissues from sample 8 (Ci Qianhu) and sample 9 (Xiong Qianhu) were showed in Figure 3 and Figure 4. A total of 34 peaks were accurately identified from all tissues, 29 compounds of which were pyranocoumarins. Peaks 15, 23, 30, and 33 were identified as imperatorin, praeruptorin A, praeruptorin B, and praeruptorin E compared with reference substances. Other compounds were confirmed by comparing the reported mass ions, retention times, and fragmentation pattern of compounds in the literature [19,20,21,22,23]. The data of the compounds are listed in Table 1.

### 2.4. Tissue-Specific Chemical Profiling of the Ci Qianhu

In this study, a total of 31 peaks were identified from the root of Ci Qianhu. Praeruptorin A (peak 23), hyuganin C (peak 24), praeruptorin E (peak 33) were detected in all microdissected tissues. Total chromatographic peaks in the cork, secondary phloem, cambium, and secondary xylem were 14, 17, 5, and 9, respectively.

Most fractures were distributed in the secondary phloem, which were roughly separated into three parts according to the number of fractures. The fractures were the most numerous on the external side, with a total of 14 peaks identified. The fractures on the middle of secondary phloem were less than those of the external, with a total of 17 peaks identified. Almost no fracture exhibited in the innermost side, with a total of 9 peaks identified.

Secretory canals were abundantly scattered in the secondary phloem, and the fluorescence intensity differed in secretory canals of various diameters. The secretory canals were divided into four types according to their sizes and fluorescence intensities. Larger and brighter secretory canals near the cork were marked as SC1. Secretory canals located on the middle of secondary phloem were smaller and showed orange-yellow and light-green fluorescence, which were marked as SC2 and SC3, respectively. Secretory canals near the cambium were smaller, which were labeled as SC4. A total of 24, 16, 11, and 8 common peaks were detected from SC1 to SC4. SC1 contained the most compounds, with the peaks 1, 3, 5, 7–9, 11, 13–16, 18–25, 28, 30–31, and 33–34 exists in SC1. In Ci Qianhu, SC1 exerted the strongest fluorescence intensity and contained the most coumarins. Meanwhile, the secondary xylem was separated into two parts, which include 9 common peaks detected at rays and 3 common peaks detected at xylem vessels and parenchymal cells.

### 2.5. Tissue-Specific Chemical Profiling of the Xiong Qianhu

A total of 31 metabolites were detected in various tissues of Xiong Qianhu, with the peaks 11, 12, 26 exclusive to Ci Qianhu while the peaks 4, 6, 10 exclusive to Xiong Qianhu. A total of 22 peaks were detected in the cork of Xiong Qianhu, which is higher than the 14 peaks in Ci Qianhu. In other samples except sample 11 and 12, the cork peaks of Xiong Qianhu were more than those of Ci Qianhu. The secondary phloem of Xiong Qianhu was narrower, with a total of 13 peaks were detected, which is less numerous by 17 than that of Ci Qianhu. A total of 21 peaks were detected in cambium of Xiong Qianhu, which was greater than that of 5 peaks of Ci Qianhu. The results showed that the secondary metabolites had a rich accumulation in cambium of Xiong Qianhu. In the secondary xylem of Xiong Qianhu, the number of fibers has increased, and the parenchymal cells decreased significantly. A total of 7 peaks were detected, which showed the least common peaks among different tissues in the root.

Xiong Qianhu exhibited fewer secretory canals with slightly larger diameters than those of Ci Qianhu. All secretory canals in the secondary phloem of Xiong Qianhu were roughly divided into two types based on their fluorescence intensity: SC1 and SC2 (see Figure 4), with 24 and 26 peaks detected, respectively.

### 2.6. Comparison Between Ci Qianhu and Xiong Qianhu

The metabolite profiling results showed that the roots of Ci Qianhu and Xiong Qianhu were mostly comprised of furanocoumarins, Ci Qianhu and Xiong Qianhu have identical results in the number of common peaks. The secretory canals of both Ci Qianhu and Xiong Qianhu contained the most coumarins, followed by the secondary phloem, and secondary xylem. In addition, the number of peaks in the cork and cambium of Xiong Qianhu were much higher than those of Ci Qianhu.

### 2.7. Quantitative Analysis Results by HPLC–DAD

Seven types of coumarins in Qianhu were detected simultaneously, including a simple coumarin (peucedanol) and three furocoumarins (xanthotoxin, bergapten, and imperatorin), and three pyranocoumarins (praeruptorin A, praeruptorin B, and praeruptorin E). The total coumarin contents of Ci Qianhu and Xiong Qianhu roots were 13.09 mg/g and 12.55 mg/g, respectively, which suggested that bolting lowered the total coumarin content of Qianhu.

Furthermore, the contents of three pyranocoumarins (praeruptorin A, praeruptorin B, praeruptorin E) in Ci Qianhu were higher than those in Xiong Qianhu, with total contents of 12.23 mg/g and 11.69 mg/g, respectively. At the same time, the contents of three furocoumarins in Ci Qianhu were slightly higher than those in Xiong Qianhu, with contents of 0.81 mg/g and 0.77 mg/g, respectively. 

In addition, as shown in Table 2, the highest levels of coumarin were found in the bark of Qianhu (29.66 mg/g and17.35 mg/g in Xiong Qianhu and Ci Qianhu, respectively), followed by in the middle part (22.71 mg/g and 16.35 mg/g in Xiong Qianhu and Ci Qianhu, respectively), with coumarin levels in the inner part as the lowest (4.10 mg/g and 5.47 mg/g in Xiong Qianhu and Ci Qianhu, respectively). These results were similar to the fluorescence microscopy results in Section 2.2.

Moreover, peucedani radix exhibits anti-inflammatory, anti-hyperlipidaemia, and anti-cancer effects [24,25], which are attributed to pyranocoumarins, such as praeruptorin A, praeruptorin B, etc. In this paper, pyranocoumarin content was lower in Xiong Qianhu than that in Ci Qianhu. Therefore, it was suggested that the medicinal efficacy of Qianhu was decreased after bolting.

## 3. Materials and Methods

### 3.1. Samples and Reagents

The healthy plants of *P. praeruptorum* Dunn (peucedani radix) were collected from Anhui Province, China. Sample information is provided in Table 3. All samples were identified by Professor Huasheng Peng (School of Pharmacy, Anhui University of Chinese Medicine). Reference substances peucedanol, imperatorin, praeruptorin A, praeruptorin B, and praeruptorin E were purchased from Baoji Chenguang Biotechnology Co., Ltd. (Shaanxi, China). Xanthotoxin were purchased from Chengdu Chroma-Biotechnology Co., Ltd. (Sichuan, China). Bergapten was purchased from Chengdu Ruifensi Biological Technology Co., Ltd. (Sichuan, China). All of the above were of >98% purity. The HPLC-grade solvent acetonitrile was obtained from TEDIA (Cincinnati, OH, USA). HPLC-grade formic acid of over 98% purity was purchased from Aladdin (Los Angeles, CA, USA). Methanol was purchased from Sinopharm Chemical Reagent Co., Ltd. (Shanghai, China). All other chemicals were of analytical grade. Water was prepared by a Direct-Pure Water System (Shanghai, China).

### 3.2. Microscopic Analysis

The fresh Ci Qianhu and Xiong Qianhu roots were cut into 0.3–0.5 cm long sections, fixed in FAA solution (70% ethanol-formaldehyde-acetic acid, 90:5:5, *v*/*v*/*v*) for 24 h, dehydrated with a series of gradient ethanol, and embedded in paraffin. The tissue block was trimmed and sliced to form 10–15 μm thick bands using a rotary microtome (Leica RM2265, Leica Microsystems, Benshein, Germany). The tissues were double-stained with safranin and Fast Green, mounted using neutral gum, and observed in normal and polarized light under a microscope (Leica DM6000B) for morphological structure.

### 3.3. UPLC-Q/TOF–MS Analysis

#### 3.3.1. Sample Preparation

The Ci Qianhu and Xiong Qianhu roots samples were prepared with the same size as in Section 2.3. Prior to sectioning, the sample was wrapped in a water-soaked noncellulosic paper at about 20 °C for 24 h. The roots were sectioned at −20 °C using a cryotome (Leica CM1850 UV; Leica Microsystems, Wetzlar Asslar, Hessen, Germany) into 30 μm thick slices and mounted on a nonfluorescent polyethylene terephthalate (PET) slide with steel frames (76 × 26 mm, 1.4 μm; Leica Microsystems). The slides were observed under a Leica LMD 7 microscope (Leica Microsystems) in fluorescence mode. Laser microdissection was performed on a Leica LMD-BGR Fluorescence Filter System at 10× magnification (20× for secretory canals and cambium) and the following conditions: aperture of 23, power of 55 μJ, speed of 83, and pulse frequency of 3217 Hz. An area of approximately 10^6^ μm^2^ was determined as the observation site and dissected separately under fluorescence inspection mode. The microdissected tissues fell into caps of 500 μL microcentrifuge tubes (Leica Microsystems) by gravity.

The microdissected tissue part in each cap was transferred to the bottom of the tube through centrifugation (Legend Micro 21R; Thermo Scientific, Waltham, MA, USA) at 12,000 rpm for 5 min. Each microcentrifuge tube was added with 100 μL methanol and subjected to ultrasound for 30 min (JK-5200B; Jinnik, Hefei, China). Each tube was centrifuged again for 10 min at 12,000 rpm. The clear supernatant was transferred to a tapered glass insert with polymer branched foot (250μL, Agilent, Santa Clara, USA) in a 1.5 mL brown HPLC vial (Agilent) and preserved at 4 °C for analysis.

#### 3.3.2. UPLC-Q/TOF–MS method

UPLC-Q/TOF–MS analysis was performed on a Waters Xevo G2-XS quadruple time-of-flight spectrometer (Waters, Milford, MA, USA) coupled with UNIFI v1.7.1 software (WatersCorp., Milford, MA, USA). Chromatographic separation was performed with the flow rate of 0.2 mL/min on an Acquity UPLC BEH C18 Column (2.1 mm × 100 mm, 1.7 μm; Waters) and C18 Pre-column (2.1×5 mm, 1.7 μm; Waters) at 25 °C. The mobile phase used was a mixture of acetonitrile (A) and 0.1% formic acid water (B). The gradient elution as follows: 0–5 min, 70–60% B; 5–10 min, 60–40 % B; 10–15 min, 40–40% B; 15–20 min, 40–34% B; 20–22 min, 34–34 % B; 22–24.5 min, 34–25% B; 24.5–27 min, 25–15% B; 27–29 min, 15–15% B; 29–31 min, 15–70% B; and 31–33 min, 70–70% B. The injection volume of the sample was 1 μL. Mass spectra were recorded over the range of *m*/*z* 50–1200 under the following conditions: capillary source of 3 kV, sampling cone source of 40, source offset of 80, source temperature of 110 °C, cone gas flow rate of 50 L h^−1^, and desolation gas flow rate of 600 L · h^−1^. Leucine enkephalin was used to calibrate the mass spectrometer.

### 3.4. HPLC–DAD Analysis

#### 3.4.1. Standard Solutions Preparation

Reference standards were dissolved in pure methanol, and the concentrations of standard solutions were made as follows: peucedanol 1.00 mg/mL, xanthotoxin 1.00 mg/mL, bergapten 0.95 mg/mL, imperatorin 0.80 mg/mL, praeruptorin A 0.98 mg/mL, praeruptorin B 0.96 mg/mL, and praeruptorin E 1.00 mg/mL. The standard solutions were passed through a 0.22 µm PTFE filter prior to HPLC analysis. The standard solution is diluted to peucedanol 10.00 ng/μL, xanthotoxin 2.00 μg/mL, bergapten 5.70 μg/mL, imperatorin 3.60 μg/mL, praeruptorin A 160.00 μg/mL, praeruptorin B 18.00 μg/mL, and praeruptorin E 18.00 μg/mL, and each component was injected according to the different volume, respectively.

#### 3.4.2. Sample Preparation

Three samples of Ci Qianhu and Xiong Qianhu were collected from Ningguo, Anhui Province, China. The Qianhu samples were separated for three parts by hand: the bark (including most of the cork), middle part (including most of the phloem and some cambium), and inner part (including most of xylem and cambium), dried to a constant weight at 40 °C, and ground into powder through a 50-mesh sieve. The dry powder (approximately 0.10 g) was mixed with 5 mL methanol, subjected to ultrasonic treatment for 30 min, and allowed to cool. Methanol was added to the mixture to compensate for the loss of weight. The mixture was subsequently filtered through a 0.45 μm microporous filter membrane and stored at 4 °C until use for HPLC analysis.

#### 3.4.3. HPLC–DAD Method

An Agilent 1260 Infinity II Diode Array Detector WR (Agilent) equipped with an Agilent 5HC- C_18_ column (4.6 mm × 250 mm, 5 μm) was used for HPLC analysis. Chromatographic separation was conducted in a flow rate of 1.0 mL/min at 35 °C with a mixture of acetonitrile (A) and water (B) as the mobile phase. The gradient elution as follows: 0–10 min, 20–40% A; 10–12 min, 40–45% A; 12–17 min, 45–80% A; 17–30 min, 80–80% A; 30–32 min, 80–20% A; and 32–34 min, 20–20% A. The injection volume of the sample was 20 μL. Samples were detected at 297 nm and 321 nm for 1–17 and 17–34 min, respectively.

#### 3.4.4. Method Validation

**Figure 5:**
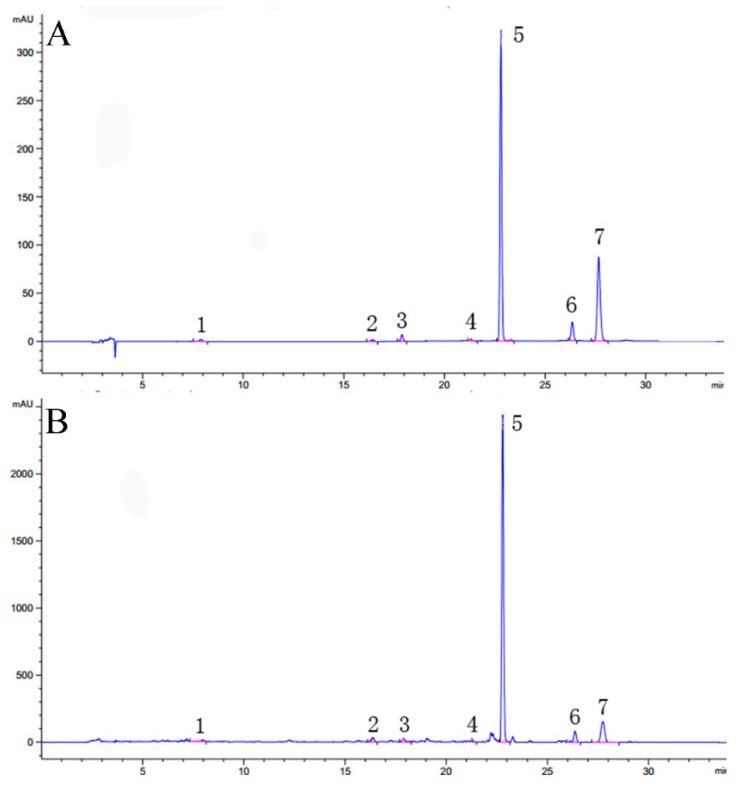
Peaks identification: (**A**) Liquid chromatogram of reference substance, (**B**) Liquid chromatogram of sample. (**1**) peucedanol; (**2**) xanthotoxin; (**3**) bergapten; (**4**) imperatorin; (**5**) praeruptorin A; (**6**) praeruptorin B; (**7**) praeruptorin E.

Method validation included linearity, repeatability, intra-precision, inter-precision, stability, recovery, limit of detection (LOD), and limit of quantitation (LOQ). Standard curves were established with 7 types of coumarins with coefficients of determination (*R*^2^) higher than 0.9972. Intra-precision and inter-precision were determined by analyzing the same control for 6 consecutive times and three day, respectively. Repeatability was determined by detecting 6 copies of the same sample. Stability was recorded at time intervals of 0 h, 2 h, 4 h, 8 h, 12 h, and 24 h for the same sample at room temperature, and the RSD results of peak area showed that the sample was stable within 24 h. A sample mixed with approximately 100 % of a known amount of the 7 types of coumarins was used for the recovery test with six replications. The LODs calculated by an S/N of 3, were 0.10 ng/μL, 0.02 ng/μL, 0.03 ng/μL, 0.02 ng/μL, 0.01 ng/μL, 0.02 ng/μL, and 0.08 ng/μL for peucedanol, xanthotoxin, bergapten, imperatorin, praeruptorin A, praeruptorin B, and praeruptorin E, respectively. LOQs calculated by an S/N of 10 were 0.22 ng/μL, 0.04 ng/μL, 0.07 ng/μL 0.06 ng/μL, 0.75 ng/μL 0.72 ng/μL, and 0.12 ng/μL, respectively. Each of the above detectable items was calculated separately for the RSD and the results were shown in Table 4, and the liquid chromatogram of reference substance and sample were shown in Figure 5.

## 4. Conclusions

In this paper, the microscopic and chemical characteristics of Qianhu were compared before and after bolting. The results showed that the roots of Qianhu began to lignify with an enlarged xylem and shrank and ruptured phloem after bolting, and the distribution of coumarins was obviously uneven in different tissues of Qianhu. The coumarins were found concentrated in the secretory canals of phloem and less in the secondary xylem. The content determination results showed that the content of coumarins in Xiong Qianhu were lower than that of Ci Qianhu, although the coumarin content presented some differences in different tissues. These results showed that coumarins accumulated differently in different tissues after bolting, and the proportion of phloem and xylem changed after bolting, which may lead to the quality difference for Ci Qianhu and Xiong Qianhu. In summary, the quality of Qianhu declined after bolting, which suggested that Xiong Qianhu was not suitable for medicinal use. This article provides data supporting the quality difference between Ci Qianhu and Xiong Qianhu, however, the genes of bolting need to be further studied to elucidate the molecular mechanism of the quality differences in between.

## Figures and Tables

**Table 1:** Chemical characterization of cross-section extractions by UPLC-QTOF–MS.

Peak Number	tR (min)	Molecular Formula	Measured Mass (m/z)	Mass Error (ppm)	MS/MS(m/z)	Identification	Existence in C/X
1	2.20	C14H14O5	263.0918	1.6	149.0234; 191.0342; 245.0808; 263.0496	Rutatin	C, X
2	2.39	C20H24O9	408.9195	−0.6	169.9816; 199.1246; 243.1513; 263.1489; 301.1412	Nodakenin	C, X
3	2.42	C10H8O4	193.0497	0.2	133.5869; 177.1078; 182.9506	Scopolin	C, X
4	2.52	C14H14O5	263.0911	2.4	107.0324; 117.0908; 135.0892; 145.0079	Khellactone	X
5	4.99	C16H16O6	305.1024	−2.9	141.9585; 175.0385; 203.0709; 245.0804; 327.0830	Qianhucoumarin B	C, X
6	6.66	C13H10O5	247.0607	2.4	189.0185; 217.0492; 232.0362; 247.0607	Isopimpinellin	X
7	6.99	C21H22O9	436.1648	0.6	175.0392; 227.0697; 245.0810; 359.1180	3′-(dihydroxyl-angel/dihydroxyl-senecioyl/dihydroxyl-tigloyl)-4′-acetylkhellactone	C, X
8	7.42	C21H22O8	425.1180	1.1	227.0705; 245.0809; 271.0971; 343.1180; 436.1979	3′-(hydroxyl-angel/hydroxyl-senecioyl/hydroxyl-tigloyl)-4′-acetylkhellactone	C, X
9	7.83	C21H22O8	425.1655	0.9	175.0392; 227.0707; 245.0815; 343.1180; 436.1936; 441.0936	3′-(hydroxyl-angel/hydroxyl-senecioyl/hydroxyl-tigloyl)-4′-acetylkhellactone	C, X
10	8.36	C18H18O7	369.0942	−0.7	175.0390; 227.0705; 245.0813; 287.0916;	Qianhucoumarin D or trans-3,4-diacetylkhellactone	X
11	8.78	C19H20O6	367.1161	2.3	147.0441; 175.0391; 203.0704; 245.0810; 345.1303	Qianhucoumarin A	C
12	9.11	C19H20O6	367.1146	0.6	175.0390; 203.0705; 245.0811; 345.1338	3′-(angeloyl/senecioyl/tigloyl)-khellactone	C
13	9.23	C21H22O8	425.1645	−0.7	203.1077; 245.0810; 327.1225; 343.1174	3′-(hydroxyl-angel/hydroxyl-senecioyl/hydroxyl-tigloyl)-4′-acetylkhellactone	C, X
14	10.46	C19H22O6	369.1307	0.4	175.0391; 203.0705; 245.0811; 329.1385; 347.1468	3′-(isovaleryl/2-methylbutyryl)- khellactone	C, X
15	10.70	C16H14O4	271.0912	−1	119.0853; 203.0337; 271.0912; 391.2840	Imperatorin	C, X
16	10.92	C19H18O6	365.0990	0	163.0388; 243.0655; 261.0763; 343.1176	Qianhucoumarin E	C, X
17	11.48	C19H20O6	367.1257	-0.1	149.0233; 243.0657; 261.0762; 345.1332	3′-(isovaleryl/2-methylbutyryl)-4′-oxo-3′,4′-dihydroseselin	C, X
18	11.54	C15H16O4	261.0762	−1	93.0567; 149.0233; 193.0492	7-methoxy-5-prenyloxycoumarin	C, X
19	11.67	C20H22O7	397.1258	0	175.0389; 199.0760; 227.0706; 245.0812; 315.1231	Hyuganin D	C, X
20	12.09	C21H22O7	409.1253	−1.4	175.0391; 245.0811; 299.1292; 327.1231	Qianhucoumarin I	C, X
21	12.22	C21H22O7	409.1258	0.1	83.0492; 175.0389; 227.0707; 327.1229	3′-(tigloyl/senecioy)-4′-acetykhellactone	C, X
22	12.37	C21H22O7	409.1257	0.5	227.0709; 245.0808; 327.1233; 404.1704; 425.0995	cis-3′-acetyl-4′-angeloykhellactone	C, X
23	12.62	C21H22O7	409.1256	−0.3	83.0498; 227.0805; 245.0808; 327.1227	Praeruptorin A	C, X
24	13.31	C21H24O7	411.1411	−0.8	175.0389; 227.0705; 245.0811; 329.1384; 411.1411	Hyuganin C	C, X
25	14.49	C22H24O7	423.1405	−2.2	175.0387; 227.0705; 245.0805; 327.1226	Qianhucoumarin J	C, X
26	15.05	C22H26O7	420.2010	−1.7	227.0705; 245.0810; 329.1384	3′-(isovaleryl/2-methylbutyryl)-4′-propionylkhellactone	C
27	17.06	C23H26O7	432.2013	−0.7	83.04914; 149.0234; 227.0704; 245.0791; 327.1228; 465.1300	3′-(angeloyl/senecioyl/tigloyl)-4-isovalerylkhellactone	C, X
28	17.48	C24H26O7	449.1517	−0.5	149.0233; 175.0387; 227.0710; 327.1226	3′-(angeloyl/senecioyl/tigloyl)-4′- (angeloyl/senecioyl/tigloyl)’-khellactone	C, X
29	17.93	C24H26O7	449.1566	−0.6	83.0493; 227.0705; 245.0806; 327.1230	3′-(angeloyl/senecioyl/tigloyl)-4′- (angeloyl/senecioyl/tigloyl)’-khellactone	C, X
30	18.23	C24H26O7	449.1563	−0.9	227.0704; 245.0808; 327.1226	Praeruptorin B	C, X
31	19.37	C24H28O7	451.1713	1.1	175.0385; 227.0706; 245.0807; 329.1386	3′-(isovaleryl/2-methylbutyryl)-4′-(tigloy/senecioyl/angeloyl)-khellactone	C, X
32	19.64	C24H28O7	451.1724	−0.6	83.0487; 199.0753; 227.0703; 327.1226	3′-(isovaleryl/2-methylbutyryl)-4′-(tigloy/senecioyl/angeloyl)-khellactone	C, X
33	20.12	C24H28O7	451.1728	0.2	83.0491; 227.0706; 245.0810; 327.1230	Praeruptorin E	C, X
34	22.07	C24H30O7	453.1886	1	175.0391; 227.2015; 245.0815; 329.1389	cis-3′,4′-diisovalerylkhellactone	C, X

**Table 2:** Amount of seven chemicals in different parts of the whole transverse section from the root of Qianhu Values are means ± SD (*n* = 6).

Category	Tissue	The Content in Each Parts (mg/g)							Total
		Peucedanol	Xanthotoxin	Bergapten	Imperatorin	Praeruptorin A	Praeruptorin B	Praeruptorin E	
**Ci Qianhu**	Bark	0.38 ± 0.17	0.09 ± 0.10	0.43 ± 0.39	0.20 ± 0.08	12.56 ± 2.45	0.84 ± 0.36	2.85 ± 1.80	17.35
	Middle part	0.09 ± 0.02	0.03 ± 0.04	0.12 ± 0.08	0.18 ± 0.10	11.76 ± 4.01	1.11 ± 0.70	3.06 ± 2.10	16.35
	Inner part	0.05 ± 0.05	0.01 ± 0.01	0.02 ± 0.01	0.10 ± 0.02	3.76 ± 3.42	0.29 ± 0.32	1.24 ± 1.46	5.46
	Whole root	0.04 ± 0.20	0.15 ± 0.20	0.43 ± 0.52	0.23 ± 0.30	8.21 ± 3.30	2.12 ± 3.24	1.90 ± 0.99	13.09
**Xiong Qianhu**	Bark	0.68 ± 0.74	0.09 ± 0.10	0.39 ± 0.34	0.31 ± 0.27	20.13 ± 4.39	1.53 ± 1.29	6.53 ± 5.14	29.65
	Middle part	0.29 ± 0.18	0.09 ± 0.13	0.40 ± 0.43	0.35 ± 0.41	15.80 ± 9.44	1.06 ± 0.82	4.72 ± 4.43	22.70
	Inner part	0.06 ± 0.00	0.02 ± 0.01	0.24 ± 0.23	0.03 ± 0.03	2.61 ± 1.52	0.21 ± 0.20	0.93 ± 0.89	4.10
	Whole root	0.09 ± 0.05	0.17 ± 0.21	0.35 ± 0.20	0.25 ± 0.29	8.62 ± 3.29	1.25 ± 1.27	1.82 ± 0.755	12.55

**Table 3:** Collection data of Qianhu.

Number	Collection Area	Collection Time	C/X (Ci Qianhu/Xiong Qianhu)	Latitude and Longitude
1 ^a^	Gangkou, Ningguo, Anhui	2016.12.20	C	30°41′42.28″ N, 118°54′51.06″ E
2 ^a^	Gangkou, Ningguo, Anhui	2017.7.12	X	30°41′42.28″ N, 118°54′51.06″ E
3 ^b^	Gangkou, Ningguo, Anhui	2016.11.16	C	30°41′42.28″ N, 118°54′51.06″ E
4 ^b^	Gangkou, Ningguo, Anhui	2017.12.10	X	30°41′42.28″ N, 118°54′51.06″ E
5 ^b^	Qimen, Huangshan, Anhui	2016.12.20	C	29°59′11.78″ N, 117°20′50.28″ E
6 ^b^	Jixi, Xuancheng, Anhui	2017.12.11	C	30°04′1.86″ N, 118°34′47.75″ E
7 ^b^	Qingyang, Chizhou, Anhui	2018.9.18	C	30°38′24.72″ N, 117°50′50.96″ E
8 ^b^	Gangkou, Ningguo, Anhui	2018.9.18	C	30°41′42.28″ N, 118°54′51.06″ E
9 ^b^	Gangkou, Ningguo, Anhui	2018.9.18	X	30°41′42.28″ N, 118°54′51.06″ E
10 ^b^	Qingyang, Chizhou, Anhui	2018.9.18	X	30°38′24.72″ N, 117°50′50.96″ E
11 ^b^	Jinde, Xuancheng, Anhui	2018.9.3	X	30°17′14.03″ N,118°32′25.62″ E
12 ^b^	Modian, Hefei, Anhui	2018.9.3	X	31°56′20.18″ N, 117°23′29.64″ E
13 ^c^	Huangdu, Ningguo, Anhui	2017.12.10	C	30°43′14.70″ N, 118°51′6.95″ E
14 ^c^	Huangdu, Ningguo, Anhui	2017.12.10	C	30°43′14.70″ N, 118°51′6.95″ E
15 ^c^	Huangdu, Ningguo, Anhui	2017.12.10	C	30°43′14.70″ N, 118°51′6.95″ E
16 ^c^	Huangdu, Ningguo, Anhui	2017.12.10	C	30°43′14.70″ N, 118°51′6.95″ E
17 ^c^	Huangdu, Ningguo, Anhui	2017.12.10	C	30°43′14.70″ N, 118°51′6.95″ E
18 ^c^	Huangdu, Ningguo, Anhui	2017.12.10	C	30°43′14.70″ N, 118°51′6.95″ E
19 ^c^	Gangkou, Ningguo, Anhui	2017.12.10	X	30°41′42.28″ N, 118°54′51.06″ E
20 ^c^	Gangkou, Ningguo, Anhui	2017.12.10	X	30°41′42.28″ N, 118°54′51.06″ E
21 ^c^	Gangkou, Ningguo, Anhui	2017.12.10	X	30°41′42.28″ N, 118°54′51.06″ E
22 ^c^	Gangkou, Ningguo, Anhui	2017.12.10	X	30°41′42.28″ N, 118°54′51.06″ E
23 ^c^	Gangkou, Ningguo, Anhui	2017.12.10	X	30°41′42.28″ N, 118°54′51.06″ E
24 ^c^	Gangkou, Ningguo, Anhui	2017.12.10	X	30°41′42.28″ N, 118°54′51.06″ E
25 ^c^	Xianyushan, Chizhou, Anhui	2018.4.21	C	29°59′10.14″ N, 117°16′57.50″ E
26 ^c^	Xianyushan, Chizhou, Anhui	2018.4.21	C	29°59′10.14″ N, 117°16′57.50″ E
27 ^c^	Xianyushan, Chizhou, Anhui	2018.4.21	C	29°59′10.14″ N, 117°16′57.50″ E
28 ^c^	Xianyushan, Chizhou, Anhui	2018.4.21	C	29°59′10.14″ N, 117°16′57.50″ E
29 ^c^	Xianyushan, Chizhou, Anhui	2018.4.21	C	29°59′10.14″ N, 117°16′57.50″ E
30 ^c^	Xianyushan, Chizhou, Anhui	2018.4.21	C	29°59′10.14″ N, 117°16′57.50″ E
31 ^c^	Xianyushan, Chizhou, Anhui	2018.8.22	X	29°59′10.14″ N, 117°16′57.50″ E
32 ^c^	Xianyushan, Chizhou, Anhui	2018.8.22	X	29°59′10.14″ N, 117°16′57.50″ E
33 ^c^	Xianyushan, Chizhou, Anhui	2018.8.22	X	29°59′10.14″ N, 117°16′57.50″ E
34 ^c^	Xianyushan, Chizhou, Anhui	2018.8.22	X	29°59′10.14″ N, 117°16′57.50″ E
35 ^c^	Xianyushan, Chizhou, Anhui	2018.8.22	X	29°59′10.14″ N, 117°16′57.50″ E
36 ^c^	Xianyushan, Chizhou, Anhui	2018.8.22	X	29°59′10.14″ N, 117°16′57.50″ E

^a^ samples were used for Paraffin section; ^b^ samples were used for Qualitative analysis; ^c^ samples were used for Quantitative analysis.

**Table 4:** Method validation of the detected chemicals.

Analytes.	Peucedanol	Xanthotoxin	Bergapten	Imperatorin	Praeruptorin A	Praeruptorin B	Praeruptorin E
Range (μg /mL)	10–60	2–100	5.7–456	3.6–360	160–16,000	18–1800	9–1440
Regression equation	y = 11.29x − 0.2129	y = 8.6057x + 5.4184	y = 24.171x − 0.2627	y = 4.9076x + 3.1037	y = 404.9x − 9.8906	y = 40.943x − 16.533	y = 190.48x + 9.9344
LOD (μg/mL)	0.10	0.02	0.03	0.02	0.01	0.02	0.08
LOQ (μg/ mL)	0.22	0.04	0.07	0.06	0.75	0.72	0.12
R2	0.9998	0.9972	1.0000	0.9998	0.9999	0.9999	1.0000
Intra-precision (%RSD)	1.85	1.67	1.04	1.46	0.95	1.29	1.14
Inter-precision (%RSD)	4.71	1.71	0.70	1.25	0.78	0.98	0.77
Repeatability (%RSD)	1.34	1.30	1.08	1.19	1.01	0.93	0.78
Stability (%RSD)	4.01	0.50	2.99	2.92	0.55	0.60	0.55
Recovery (%)	95.54	100.25	99.18	95.45	102.30	98.98	102.99

## References

[1] Chang H.T., Okada Y., Okuyama T., Tu P.F. (2007). ^1^H and ^13^C NMR assignments for two new angular furanocoumarin glycosides from *Peucedanum praeruptorum*. Magn. Reson. Chem..

[2] Kim H.M., Jeong S.Y., Kim S.M., Lee K.H., Kim J.H., Seong R.S. (2016). Simultaneous Determination and Recognition Analysis of Coumarins in *Angelica decursiva* and *Peucedanum praeruptorum* by HPLC-DAD. Nat. Prod. Sci..

[3] Takata M., Shibata S., Okuyama T. (1990). Structures of Angular Pyranocoumarins of Bai-Hua Qian-Hu, the Root of *Peucedanum praeruptorum*. Planta Med..

[4] Chen L.L., Zhang L., Fang Q.Y., Chu S.S., Peng H.S., Dai M. (2018). Factors influencing the quality of *Peucedanum praeruptorum* Dunn and “assessment of quality based on its features”. Chin. J. Med. Hist..

[5] Lee A.R., Jin M.C., Lee A.Y., Kim H.S., Gu G.J., Kwon B.I. (2017). Reduced allergic lung inflammation by root extracts from two species of Peucedanum through inhibition of Th2 cell activation. J. Ethnopharmacol..

[6] Lee J., Lee Y.J., Kim J., Bang O.S. (2015). Pyranocoumarins from Root Extracts of *Peucedanum praeruptorum* Dunn with Multidrug Resistance Reversal and Anti-Inflammatory Activities. Molecules.

[7] Liu X.Q., Chin J.F., Qu X.H., Bi H.D., Liu Y., Yu Z.Q., Zhai Z.J., Qin A., Zhang B., Dai M. (2017). The Beneficial Effect of Praeruptorin C on Osteoporotic Bone in Ovariectomized Mice via Suppression of Osteoclast Formation and Bone Resorption. Front. Pharmacol..

[8] Huang L., Wu Q., Li Y.H., Wang Y.T., Bi H.C. (2013). PXR-Mediated Upregulation of CYP3A Expression by Herb Compound Praeruptorin C from *Peucedanum praeruptorum* Dunn. Evid. Based Complement. Altern. Med..

[9] Hou Z.G., Xu D.R., Yao S., Luo J.G., Kong L.Y. (2009). An application of high-speed counter-current chromatography coupled with electrospray ionization mass spectrometry for separation and online identification of coumarins from *Peucedanum praeruptorum* Dunn. J. Chromatogr. B Anal. Technol. Biomed. Life Sci..

[10] An K., Xie T., Zhu D., Dong Y., Wen H.M., Pei Y.Q., Shan J.J., Di L.Q. (2017). Comparative pharmacokinetic study of pyranocoumarins and khellactone in normal and acute lung injury rats after oral administration of *Peucedanum praeruptorum* Dunn extracts using a rapid and sensitive liquid chromatography-tandem mass spectrometry method. Biomed. Chromatogr..

[11] Song Y., Jing W., Yan R., Wang Y. (2015). Research progress of the studies on the roots of *Peucedanum praeruptorum* dunn (*Peucedani radix*). Pak. J. Pharm. Sci..

[12] Yu N.J., Wu W.L., Liu S.J., Fang C.W., Xiang J.R., Pan L. (2013). Dynamic accumulation of dry substance and active components in root of *Peucedanum praeruptorum*. China J. Chin. Mater. Med..

[13] Chen C.W., Han B.X. (2013). Influence of Early Bolting on the Chemical Compositions of Radix Peucedani Root by LC-MS. J. Food. Sci. Biotechnol..

[14] Mao J., Han B.X. (2010). Analysis on the Effect of Flowering on Chemical Constituents of *Peucedanum praeruptorum* Dunn Based on HS-GC-MS. J. Anhui Agric. Sci..

[15] Zhou W.W., Liang Z.T., Li P., Zhao Z.Z., Chen J. (2018). Tissue-specifc chemical profling and quantitative analysis of bioactive components of *Cinnamomum cassia* by combining laser-microdissection with UPLC-Q/TOF–MS. Chem. Cent. J..

[16] Xie W.J., Zhang H.J., Zeng J.G., Chen H.B., Zhao Z.Z., Liang Z.T. (2016). Tissues-based chemical profiling and semi-quantitative analysis of bioactive components in the root of *Salvia miltiorrhiza* Bunge by using laser microdissection system combined with UPLC-q-TOF-MS. Chem. Cent. J..

[17] Zuo Z., Zheng Y.J., Liang Z., Liu Y., Tang Q., Liu X., Zhao Z.Z. (2017). Tissue-specific metabolite profiling of benzylisoquinoline alkaloids in the root of Macleaya cordata by combining laser-microdissection with ultra-high performance liquid chromatography tandem mass spectrometry. Rapid Commun. Mass Spectrom..

[18] Wang K.P., Lei Y., Chen J.P., Ge Z.H., Liu W., Zhang Q., Chen S.J., Hu Z.Q. (2018). The coumarin conjugate: Synthesis, photophysical properties and the ratiometric fluorescence response to water content of organic solvent. Dyes Pigment..

[19] Jiang X.J. (2013). Chemical Components Analysis of Radix Peucedani by HPLC-MS and Library Searching Technologies. Master’s Thesis.

[20] Xu Q., Hu Y.F., Wang D.L., Xu G.B., Wang N. (2015). Analysis on Peucedani Radix coumarin by UPLC/Q-TOF MS and study on its preliminary pharmacodynamics. Chin. Trad. Herb. Drugs.

[21] Tian Y.L. (2016). Study on quality control method of Peucedani Radix and differentiation of furanocoumarin isomers by mass spectrometry. Master’s Thesis.

[22] Zhao Y.C., Liu T.T., Luo J., Zhang Q., Xu S., Han C., Xu J.F., Chen M.H., Chen Y.J., Kong L.G. (2015). Integration of a Decrescent Transcriptome and Metabolomics Dataset of *Peucedanum praeruptorum* to Investigate the CYP450 and MDR Genes Involved in Coumarins Biosynthesis and Transport. Front. Plant. Sci..

[23] Song Y.L., Jing W.H., Du G., Yang F.Q., Yan R., Wang Y.T. (2014). Qualitative analysis and enantiospecific determination of angular-type pyranocoumarins in Peucedani Radix using achiral and chiral liquid chromatography coupled with tandem mass spectrometry. J. Chromatogr. A.

[24] Wu M.H., Lin C.L., Chiou H.L., Yang S.F., Lin C.Y., Hsieh Y.S., Liu C.J. (2018). Praeruptorin A Inhibits Human Cervical Cancer Cell Growth and Invasion by Suppressing MMP-2 Expression and ERK1/2 Signaling. Int. J. Mol. Sci..

[25] Zheng Z.G., Lu C., Thu P.M., Zhang X., Li H.J., Li P., Xu X.J. (2018). Praeruptorin B improves diet-induced hyperlipidemia and alleviates insulin resistance via regulating SREBP signaling pathway. RSC Adv..

